# Ablation of NMDA Receptors Enhances the Excitability of Hippocampal CA3 Neurons

**DOI:** 10.1371/journal.pone.0003993

**Published:** 2009-01-14

**Authors:** Fumiaki Fukushima, Kazuhito Nakao, Toru Shinoe, Masahiro Fukaya, Shin-ichi Muramatsu, Kenji Sakimura, Hirotaka Kataoka, Hisashi Mori, Masahiko Watanabe, Toshiya Manabe, Masayoshi Mishina

**Affiliations:** 1 Department of Molecular Neurobiology and Pharmacology, Graduate School of Medicine, University of Tokyo, Tokyo, Japan; 2 Division of Neuronal Network, Institute of Medical Science, University of Tokyo, Tokyo, Japan; 3 Department of Anatomy, Hokkaido University School of Medicine, Sapporo, Japan; 4 Division of Neurology, Department of Medicine, Jichi Medical University, Tochigi, Japan; 5 Department of Cellular Neurobiology, Brain Research Institute, Niigata University, Niigata, Japan; 6 CREST, JST, Kawaguchi, Japan; The University of Queensland, Australia

## Abstract

Synchronized discharges in the hippocampal CA3 recurrent network are supposed to underlie network oscillations, memory formation and seizure generation. In the hippocampal CA3 network, NMDA receptors are abundant at the recurrent synapses but scarce at the mossy fiber synapses. We generated mutant mice in which NMDA receptors were abolished in hippocampal CA3 pyramidal neurons by postnatal day 14. The histological and cytological organizations of the hippocampal CA3 region were indistinguishable between control and mutant mice. We found that mutant mice lacking NMDA receptors selectively in CA3 pyramidal neurons became more susceptible to kainate-induced seizures. Consistently, mutant mice showed characteristic large EEG spikes associated with multiple unit activities (MUA), suggesting enhanced synchronous firing of CA3 neurons. The electrophysiological balance between fast excitatory and inhibitory synaptic transmission was comparable between control and mutant pyramidal neurons in the hippocampal CA3 region, while the NMDA receptor-slow AHP coupling was diminished in the mutant neurons. In the adult brain, inducible ablation of NMDA receptors in the hippocampal CA3 region by the viral expression vector for Cre recombinase also induced similar large EEG spikes. Furthermore, pharmacological blockade of CA3 NMDA receptors enhanced the susceptibility to kainate-induced seizures. These results raise an intriguing possibility that hippocampal CA3 NMDA receptors may suppress the excitability of the recurrent network as a whole *in vivo* by restricting synchronous firing of CA3 neurons.

## Introduction

Hippocampal CA3 pyramidal neurons form abundant recurrent connections with other CA3 neurons [1], [2]. The activity of single pyramidal neurons spreads to other CA3 neurons and this facilitates the rapid synchronization of action-potential firing in CA3 neurons [3]. Synchronized discharges of hippocampal CA3 neurons are supposed to underlie network oscillations [4], memory consolidation [5] and seizure generation [6]. Physiological sharp wave (SPW) activity that occurs during slow-wave sleep and behavioral immobility is dependent on synchronous discharges by population of CA3 pyramidal neurons [7], [8]. Synchronized CA3 activity may also contribute to the pathological EEG pattern, known as an interictal spike, which indicates a propensity for temporal lobe seizures [6].

NMDA receptors play key roles in synaptic plasticity and memory [9]. In the CA3 network, NMDA receptors are abundant at the commissural/associational synapses but scarce at the mossy fiber synapses [10]. Thus, the CA3 recurrent network is under the control of NMDA receptors. NMDA receptors in the hippocampal CA3 region are implied in rapid acquisition and recall of associative memory as well as paired associate learning [11]–[13]. On the other hand, studies with hippocampal slices showed that the synchronous network activity induces NMDA receptor-dependent LTP of CA3 recurrent synapses [14] and that stimuli that induced NMDA receptor-dependent LTP in the CA3 region generated sharp wave-like synchronous network activity [15]. These *in vitro* observations raised the hypothesis that the NMDA receptor-mediated LTP contributes to the generation of synchronous network activity. Here, we generated hippocampal CA3 pyramidal neuron-specific NMDA receptor mutant mice on the pure C57BL/6N genetic background. The ablation of hippocampal CA3 NMDA receptors resulted in the enhancement of the susceptibility to kainate-induced seizure and the emergence of characteristic large EEG spikes. We also showed that the virus-mediated ablation of hippocampal CA3 NMDA receptors in the adult mice generated characteristic large EEG spikes and that pharmacological blockade of CA3 NMDA receptors enhanced the susceptibility to kainate-induced seizures. These results raise an intriguing possibility that NMDA receptors may control negatively the excitability of the hippocampal CA3 recurrent network as a whole *in vivo*.

## Methods

### Generation of mice

Genomic DNA carrying the exon 11 to 22 of the *GluRζ1* gene was isolated by screening a bacterial artificial chromosome (BAC) library prepared from the C57BL/6 strain (Incyte Genomics) with the 2.2 kb-*Eco*RI fragment from pBKSAζ1 [16]. The 13.3-kb *Eco*RI-*Xba*I fragment of the BAC clone was used for construction of the targeting vector. The *loxP* site was inserted into the *Bam*HI site between exon 18 and 19, and the 1.8-kb DNA fragment carrying the *loxP* sequence and *Pgk-1* promoter-driven neomycin phosphotransferase gene (*neo*) flanked by two Flp recognition target (*frt*) sites into the *Spe*I site between exon 20 and 21. Endogenous *Eco*RI site at the 5′ end of 13.3-kb *Eco*RI-*Xba*I genomic fragment was replaced with *Not*I site and an exogenous *Eco*RI site was inserted between the second *loxP* site and *neo* gene. The targeting vector pζ1TV was composed of the 14.8-kb *Not*I-*Xba*I fragment, MC1 promoter-driven diphteria toxin gene derived from pMC1DTpA and pBluescript II SK(+) [17]. The targeting vector was linearized by *Not*I and electroporated into ES cells derived from the C57BL/6N strain [18], [19]. Recombinant clones were identified by Southern blot analysis of *Eco*RI-digested genomic DNA using 284-bp fragment amplified with primers 5′-ATAGAGAAAGACATGGGGC-3′ and 5′-TGCTACTGTGCAGGAAGTG-3′ from pζ1TV, the 0.6 kb *Pst*I fragment from pLFNeo [20], and the 1.1-kb *Xho*I–*Eco*RI fragment from the BAC clone as 5′ inner, neo, and 3′ outer probes, respectively. The *GluRζ1* ^flox^ allele was also identified by PCR using primers 5′-GCAGTGAGGCTCACACAGGCCTGAAGACTA-3′ and 5′-AGTGAACTCGGATCCTGACCATTGGCCACT-3′. Chimeric mice production and elimination of the *neo* gene from the genome through Flp/frt-mediated excision were carried out essentially as described [18]–[20].

**Figure 1:**
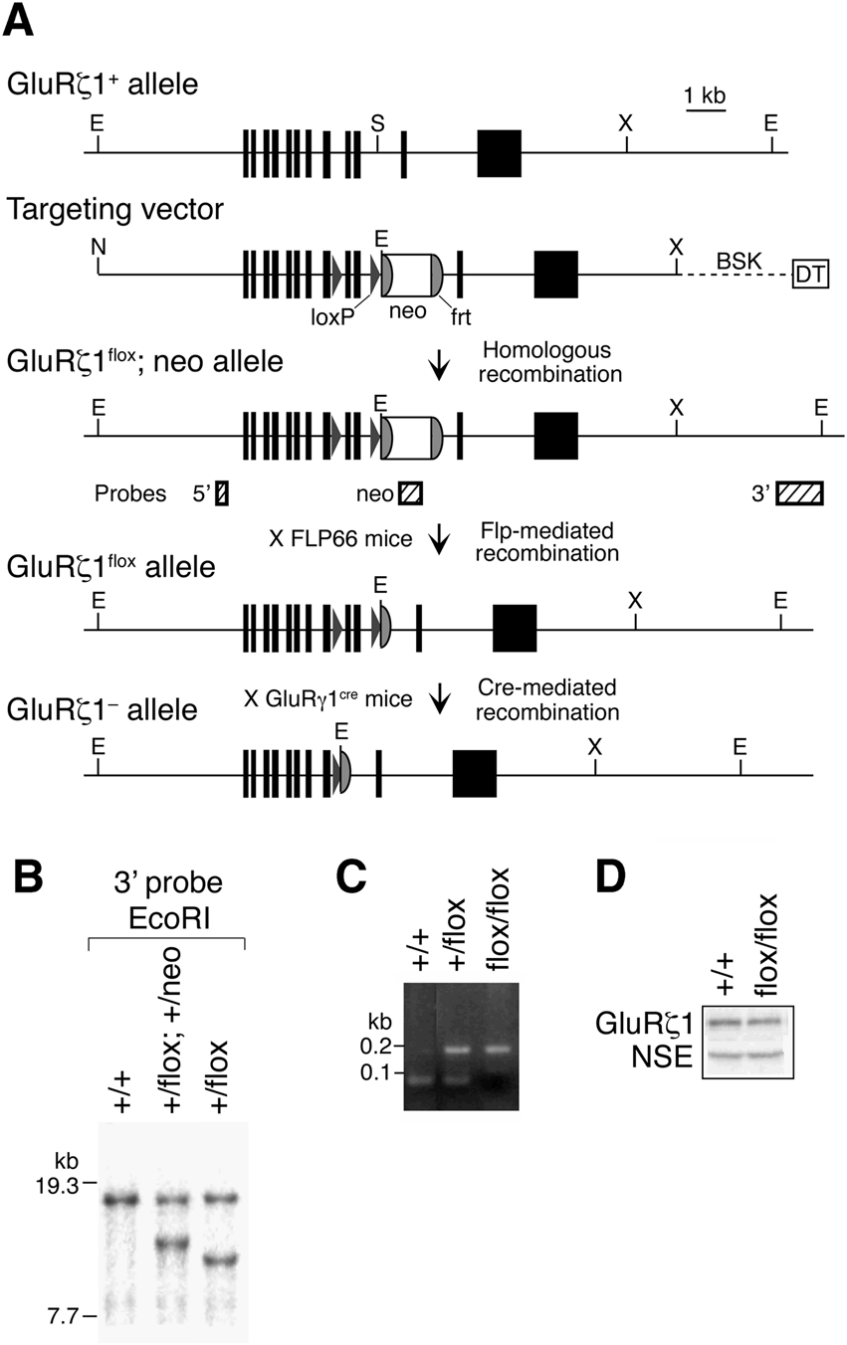
Generation of *GluRζ1* ^flox^ mice by homologous recombination in C57BL/6 strain derived ES cells. A, Schema of the exons 11–22 region of the *GluRζ1* gene (*GluRζ1* ^+^), targeting vector, floxed and *neo*-inserted allele (*GluRζ1* ^flox^; *neo*), and floxed allele (*GluRζ1* ^flox^). Exons 19 and 20 encode the putative transmembrane segment M4 of GluRζ1. The *GluRζ1* ^flox^; *neo* allele contains two *loxP* sequences flanking exons 19 and 20 of the *GluRζ1* gene and the *neo* gene flanked by two *frt* sequences. The *neo* gene was removed *in vivo* by crossing *GluRζ1* ^+/flox^; +/*neo* mice with FLP66 mice carrying the Flp recombinase gene under the control of the *EF1α* promoter. *GluRζ1* ^+/flox^ mice were crossed with GluRγ1-Cre mice to disrupt the GluRζ1 gene selectively in the hippocampal CA3 region. Abbreviations: BSK, plasmid pBluescript; DT, diphtheria toxin gene; neo, neomycin phosphotransferase gene; E, *Eco*RI; N, *Not*I; S, *Spe*I; X, *Xba*I. Hatched boxes indicate the location of probes for Southern blot analysis. B, Southern blot analysis of genomic DNA from *GluRζ1* ^+/+^, *GluRζ1* ^+/flox^; +/*neo*, and *GluRζ1* ^+/flox^ mice. *Eco*RI-digested DNA was hybridized with 3′ probe. C, Agarose gel electrophoresis of DNA fragments amplified by PCR from *GluRζ1* ^+/+^, *GluRζ1* ^+/flox^ and *GluRζ1* ^flox/flox^ mice. The amplified DNA fragments derived from the *GluRζ1* ^+^ and *GluRζ1* ^flox^ alleles were 61 bp and 169 bp, respectively. D, Western blot analysis of GluRζ1 and neuron-specific enolase (NSE) proteins in whole-brain homogenates from *GluRζ1* ^+/+^ and *GluRζ1* ^flox/flox^ mice.

GluRγ1-Cre mice were obtained by inserting the *cre* gene in the translational initiation site of the *GluRγ1* gene in frame using ES cells derived from the C57BL/6N strain [19]. The 1.8-kb DNA fragment, which carried the polyadenylation signal sequence and *pgk-1* promoter-driven *neo* gene flanked by two *frt* sites [20], was inserted into the downstream of the *cre* gene. *GluRζ1* ^+/flox^ mice were crossed with GluRγ1-Cre mice to yield *GluRγ1* ^+/cre^; *GluRζ1* ^flox/flox^ mice. The *GluRγ1* ^+/cre^ allele was identified by PCR using primers 5′-AACTGCAGTCTTGCATGCTCTCTGGAGCC-3′, 5′-GGAGCGGAGACACGGGGCAT-3′ and 5′-TTGCCCCTGTTTCACTATCC-3′. Cre recombinase-mediated NMDA receptor ablation is hippocampal CA3 pyramidal neuron-specific in *GluRγ1* ^+/cre^; *GluRζ1* ^flox/flox^ mice (Fig. 1). It is unknown why the *GluRγ1* promoter-driven Cre expression does not exactly follow the expression pattern of GluRγ1 [21]. The insertion of the *pgk-1* promoter-driven *neo* gene and the polyadenylation signal sequence together may affect the Cre expression pattern since the elimination of the *neo* gene through Flp-mediated recombination altered the expression pattern.

All animal procedures were approved by the Animal Care and the Use Committee of Graduate School of Medicine, the University of Tokyo (Approval # 1721T062). Mice were fed *ad libitum* with standard laboratory chow and water in standard animal cages under a 12 h light/dark cycle.

### AAV-Cre vector

We employed AAV to deliver Cre recombinase since AAV is safe, non-pathogenic, non-inflammatory and extremely stable stable [22], [23]. AAV-Cre or AAV-EGFP vector contains an expression cassette consisting of a human cytomegalovirus immediate-early promoter (CMV promoter), followed by the human growth hormone first intron, cDNA of Cre recombinase with a nuclear localization signal or the enhanced green fluorescence protein (EGFP), and simian virus 40 polyadenylation signal sequence (SV40 polyA), between the inverted terminal repeats (ITR) of the AAV-2 genome. The two helper plasmids, pAAV-RC and pHelper (Agilent Technologies, Santa Clara, California), harbor the AAV *rep* and *cap* genes, and the *E2A*, *E4*, and *VA RNA* genes of the adenovirus genome, respectively. HEK293 cells were cotransfected by the calcium phosphate coprecipitation method with the vector plasmid, pAAV-RC, and pHelper. AAV vectors were then harvested and purified by two sequential continuous iodoxale ultracentrifugations. The vector titer was determined by quantitative DNA dot-blot hybridization or quantitative PCR of DNase-I-treated vector stocks. Before administration, AAV vectors were diluted in phosphate-buffered saline to 5–8×10^10^ genome copies/µl. A glass micropipette was inserted into the hippocampal CA3 region of ketamine-anesthetized mice (AP, L, V = −1.2, ±1.2, +2.0; −1.7, ±2.0, +2.1; −2.2, ±2.5, +2.4; −2.7, ±3.2, +3.5; −3.2, ±2.5, +4.0). Two minutes after the insertion, 1.0 µl of a virus solution or vehicle was injected at a constant flow rate of 16.6 nl/min, and the glass micropipette was left in this configuration for an additional 2 min, to prevent reflux of the injected material along the injection track, before being slowly retracted. AAV spread 0.5–0.7 mm both rostrodorsally and laterally. For every injected animal, the limit of the infected region was verified by immunohistochemistry for Cre recombinase or GluRζ1.

### Immunological analysis

Immunohistochemistry was done as described [24] using antibodies against VGluT2 (guinea pig) [25], Calbindin (rabbit) [26], PSD-95 (rabbit) [27], GluRα1 (rabbit) [28], GAD (guinea pig) [29], and Cre recombinase (1∶1000; rabbit; Novagen). Immunobloting analyses in whole-brain homogenate were carried out using antibodies for GluRζ1 (rabbit) [30], and neuron-specific enolase (1∶4000; Chemicon) and chemiluminescense (Amersham Biosciences).

### Golgi staining

Coronal brain sections (2 mm) were immersed for 4 days in a solution composed of 5% glutaraldehyde (Wako) and 2% K_2_Cr_2_O_7_ (Sigma) and then transferred to a 0.75% solution of AgNO_3_ (Sigma) for further 4 days. The treated brain was sectioned (100 µm), dehydrated and mounted on glass slides.

### Morphology of AAV-EGFP infected CA3 neurons

AAV-EGFP vector was delivered into the hippocampal CA3 region of ketamine-anesthetized control and mutant mice of 8 weeks old. Fourteen days later, fixed coronal brain sections (150 µm) were prepared. Neurons were examined with a Leica SP-5 confocal laser scanning microscope. Optical sections were collected at intervals of 0.15 µm and averaged 16 times using a 100×objective (N.A. 1.4). The distance between axonal varicosities was measured from 50 µm-portions of CA3 axons within the CA3 stratum radiatum [31]. For spine analysis, only spines on clearly visible tertiary apical and basal branches were imaged. During the quantitation of the spine density, putative spines in the three-dimensional reconstructed image were compared with both the unprocessed, individual optical sections and with a ‘movie’, in which segments of the three-dimensional reconstruction were rotated around the dendritic axis (IMARIS, Bitplane). For dendritic analysis, neurons were imaged on a Leica SP-5 with a 40×objective (N.A. 0.8). Optical sections were collected at intervals of 2 µm and averaged 8 times. The topographical order of the dendritic tree was made using the semi-automated program FilamentTracer (Bitplane). Analysis of dendritic topology included dendritic branches up to the third order. Analysis of dendritic spines was performed in rather linear, apical secondary and tertiary dendrites.

### *In situ* hybridization

Isotopic detection of mRNAs was performed as described [32]. All samples were subjected to hybridization analysis at the same time and sections were exposed to a single x-ray film for measurement of relative optical density with IP Lab software. The relative expression levels of the mRNAs in the hippocampal CA3 region were calculated using the ratio of the density in the CA3 region to that of the CA1 region, except that the *GluRγ1* mRNA density in the CA3 region was directly compared between control and mutant mice. Double *in situ* hybridization was performed with mixture of [^33^P]dATP-labeled oligonucleotide probe for GluRζ1 (complementary to residues 2583–2627, GenBank accession No. D10028) and digoxigenin (DIG)-labeled cRNA probe for GAD67 (complementary to residues 802–1617, No. A28072) as described [33]. Hybridization signals were visualized with nuclear track emulsion (NTB-2, Kodak) and fluorescent substrate (HNPP Fluorescent Detection Set, Boehringer-Mannheim), respectively. Sections were counterstained with NeuroTrace 500/525 green (Molecular Probes).

### Kainate-induced seizure

Kainate was intraperitoneally administered to mice, and they were monitored for 1 h to determine whether they exhibited seizures with generalized tonic-clonic activity accompanying the loss of postural tone. Mice were then fixed under deep pentobarbital anesthesia for immunohistochemical analysis with the c-Fos antibody (Oncogene) 2 h after kainate administration.

### Electrophysiology

Transverse hippocampal slices (400 µm thick) were superfused with an artificial cerebrospinal fluid (aCSF) containing (in mM): 119 NaCl, 2.5 KCl, 2.5 CaCl_2_, 1.3 MgSO_4_, 1 NaH_2_PO_4_, 26.2 NaHCO_3_, and 11 glucose, which was equilibrated with 95% O_2_/5% CO_2_. Synaptic responses were evoked via a bipolar stimulating electrode placed in the CA3 stratum radiatum and whole-cell recordings were made from CA3 pyramidal cells using the blind-patch technique. The stimulus strength was set at the beginning of each experiment so that the average amplitude of synaptic responses in the absence of any antagonists is around 200 pA at a holding potential of −80 mV. The AMPA receptor-mediated excitatory postsynaptic current (AMPA-EPSC) was isolated by subtracting the synaptic response in the presence of 10 µM 6-cyano-7-nitroquinoxaline-2,3-dione (CNQX) from that in its absence. The NMDA receptor-mediated excitatory postsynaptic current (NMDA-EPSC) was recorded at +50 mV in the presence of 10 µM CNQX and 0.1 mM picrotoxin. The GABA_A_ receptor-mediated inhibitory postsynaptic current (GABA_A_-IPSC) was recorded at 0 mV in the presence of 10 µM CNQX and 25 µM D-2-amino-5-phosphonovaleric acid (D-APV). The stimulus strength was constant throughout each experiment. The slow hyperpolarizing currents induced by high-frequency stimulation (50 Hz, 40 pulses) were recorded at −20 mV in the presence of 0.1 mM picrotoxin as described previously [34]. Patch electrodes were filled with an internal solution containing (in mM): 140 potassium methansulfonate, 8 NaCl, 10 HEPES, 2 MgATP, and 0.3 Na_3_-GTP (pH 7.2 adjusted with KOH, osmolarity 290 to 300 mOsm). For pharmacological experiments, 10 mM BAPTA was added in the pipette solution or potassium methansulfonate in the pipette solution was replaced by cesium methansulfonate. Voltage-clamped responses were recorded with an Axopatch 1D amplifier (Axon Instruments, Union City, CA, USA) and the signal was filtered at 1 kHz, digitized at 2.5 kHz, and stored on a personal computer.

### Field potential recording *in vivo*

Urethane-anesthetized mice (1 g/kg body weight, i.p.) were fixed in a stereotaxic head holder (Narishige). For the recording of local field potentials, a tungsten electrode (2–5 MΩ, Frederick Haer) or a silicon probe (16 recoding sites with 50 µm separation, NeuroNexus Technologies) was inserted into the hippocampal CA3 region (AP = −2.0 mm from bregma, L = ±2.3 mm from midline, and V = +2.0 mm ventral to dura), the hippocampal CA1 region (AP = −2.0, L = ±1.0, V = +1.2) or the dentate gyrus (AP = −2.0, L = ±1.0, V = +2.0). Signals were amplified (MEG-1200, Nihon Kohden), band-pass filtered (0.08–1,000 Hz), digitized at 1 kHz through an AD converter (National Instruments), and stored in a computer. Analyses of data were performed offline using LabVIEW (National Instruments) and IGOR (Wave matics) software. Recordings using a glass electrode (10–15 MΩ, GD-2, Narishige) were carried out as described [35]. Raw traces (0.08–3,000 Hz) were band-pass filtered for the detection of MUA of neurons (0.15–3 kHz). EEG spikes with power of twice the s.d. from the baseline mean and the duration of about 30 ms were extracted. The unit activity was defined as a power of more than five times the s.d. from the baseline mean and the duration of less than 4 ms [7]. The locations of the electrode were verified histologically. CSD analyses were carried out as described [8].

#### Pharmacological experiments

Mice were anesthetized with ketamine (80 mg/kg, i.p.; Sankyo Co., Tokyo, Japan) and xylazine (20 mg/kg i.p.; Bayer, Tokyo, Japan), and fixed to a stereotaxic apparatus (David Kopf, Tujunga, CA, USA). Two single guide cannulae (Plastics One, Roanoke, VA, USA) were implanted into the CA3 region of the hippocampus bilaterally (stereotaxic coordinates: AP = −2.2 mm from bregma, ML = ±2.5 mm from midline, DV = +1.4 mm from bregma), according to an atlas of the mouse brain [36]. The tip of the internal cannula for microinjection was inserted 1 mm below the tip of the guide cannulae (DV = +2.4 mm from bregma). The cannulae were fixed to the skull with dental cement. The animals were allowed to recover for at least 5 days. D,L-APV (Sigma-Aldrich, MO, USA) was dissolved in aCSF at a concentration of 30 mM. The aCSF was consisted of NaCl (150 mM), KCl (3 mM), CaCl_2_ (1.4 mM), MgCl_2_ (0.8 mM), Na_2_HPO_4_ (0.8 mM), and NaH_2_PO_4_ (0.2 mM). During drug infusions, the mice were restrained lightly in the disposable vinyl jacket (Braintree Scientific, Inc, MA, USA) and 0.5 µl of the drug or aCSF was infused at a rate of 0.2 µl/min using a microinjection pump (CMA/100, CMA/Microdialysis, Solna, Sweden). The infusion cannulae (bilateral) were left in place for a further 1 min to diffuse the drug from the needle tip, and the animal was then returned to its home cage. Kainate was delivered i.p. 20–30 min after APV injection.

### Statistical analysis

All behavioral experiments were performed in a blind fashion. Data were expressed as mean±SEM. Statistical analysis was performed using Fisher's exact probability test, Kolmogorov-Smirnov test, log-rank test and Student *t*-test as appropriate. Statistical significance was set at *p*<0.05.

## Results

### Selective ablation of NMDA receptors in hippocampal CA3 pyramidal neurons

We disrupted the NMDA receptor *GluRζ1/NR1* gene specifically in the hippocampal CA3 pyramidal cells by Cre-*loxP* recombination on the C57BL/6N genetic background. By crossing a target mouse line carrying two *loxP* sequences flanking exon 19 and 20 of the *GluRζ1* gene (*GluRζ1* ^+/flox^ mice) with a hippocampal CA3 region-dominant Cre mouse line carrying the Cre recombinase gene inserted into the *GluRγ1/KA-1* gene (GluRγ1-Cre mice), we obtained *GluRγ1* ^+/cre^; *GluRζ1* ^flox/flox^ mice and *GluRζ1* ^flox/flox^ mice (Fig. 1), and used them in subsequent experiments as mutant and control mice, respectively.

**Figure 2:**
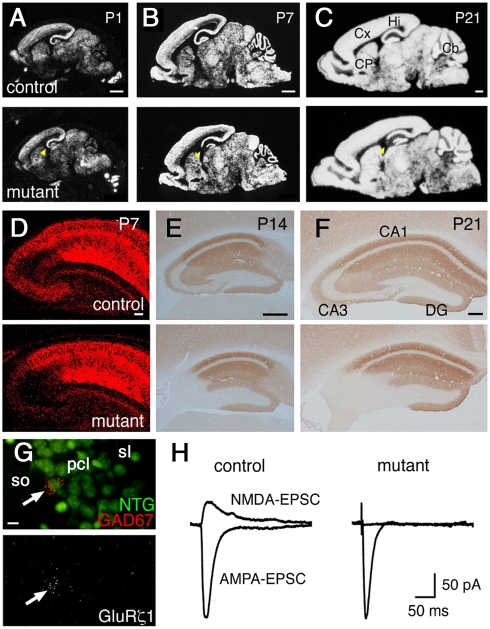
Generation of CA3 pyramidal neuron-selective NMDA receptor knockout mice. A–C, X-ray film autoradiography for *GluRζ1* mRNAs. Arrowheads indicate the CA3 region. D–F, Immunohistochemistry for GluRζ1 proteins. G, Double *in situ* hybridization for *GluRζ1* (white) and *GAD67* mRNA (red), counterstained with neurotrace green (green), in the mutant CA3 region. Arrow indicates a neuron expressing both *GluRζ1* and *GAD67* mRNAs. Scale bars: A–C, 1 mm; D–F, 200 µm; G, 10 µm. Abbreviations: Cb, cerebellum; CP, caudate-putamen; Cx, cortex; DG, dentate gyrus; Hi, hippocampus; pcl, pyramidal cell layer; sl, stratum lucidum; so, stratum oriens. H, Representative traces of AMPA- and NMDA-EPSCs at CA3 commissural/associational synapses.

*In situ* hybridization signals for the *GluRζ1* mRNA were indistinguishable between mutant and control mice at postnatal day 1 (P1) (Fig. 2A). At P7, *GluRζ1* signals were diminished specifically in the hippocampal CA3 region of mutant mice (Fig. 2B). At P21 to P23, the hybridization signals were hardly detectable in the CA3 region of mutant mice and slightly decreased in the brainstem (Fig. 2C). Residual hybridization signals for the *GluRζ1* mRNA were co-localized with those of the *GAD67* mRNA, suggesting that expression of the *GluRζ1* mRNA was intact in CA3 interneurons (Fig. 2G, *n* = 17 out of 17 GAD67-positive cells). Immunohistochemical analyses showed that immunoreactivity for GluRζ1 protein was present in the CA3 region at P7, though the amount appeared to be decreased (Fig. 2D). However, the expression of GluRζ1 protein was diminished to a negligible level at P14 and P21 (Fig. 2E and F).

We examined NMDA-EPSCs by whole-cell patch-clamp recordings from the pyramidal cell in the CA3 region of the hippocampus at P21 to P23. NMDA-EPSCs were evoked by stimulating associational/commissural fibers that mainly terminate in the stratum radiatum since NMDA receptors are more abundantly expressed in the stratum radiatum than in the stratum lucidum (Fig. 2H). In mutant mice, NMDA-EPSCs were not detectable, while AMPA-EPSCs were normally evoked. The ratios of the amplitudes of NMDA-EPSCs to those of AMPA-EPSCs were 50.9±16.1% (mean±s.e.m.) in control mice and 0.2±0.2% in mutant mice (*n* = 4 each; *t*-test, *P* = 0.03). Thus, NMDA receptors were abolished in hippocampal CA3 pyramidal neurons of mutant mice by P21. We used mutant and control mice at P21 to P23 in the following experiments unless otherwise specified.

### Enhanced susceptibility of mutant mice to kainate-induced seizure

**Figure 3:**
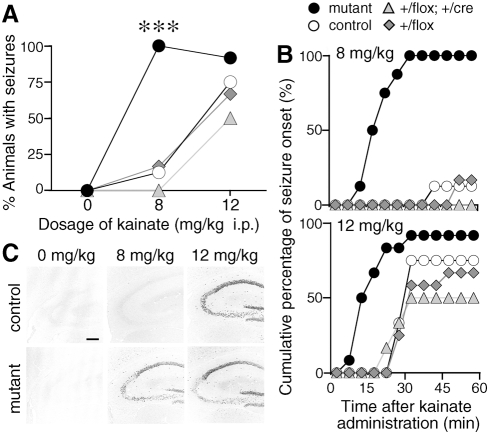
Increased susceptibility to kainate-induced tonic-clonic seizures in the mutant mice. A, The graph represents the percentage of mice with the generalized tonic-clonic seizures 1 h after drug administration. ***, *P*<0.001, Fisher's exact probability test. B, Cumulative curves for the onset of seizure. Saline, *n* = 4–6; 8 mg/kg, *n* = 7–8; 12 mg/kg, *n* = 12. C, c-Fos immunohistochemistry in the hippocampus. Scale bar, 200 µm.

To monitor the excitability of CA3 recurrent circuits *in vivo*, we tested the kainate sensitivity of mutant mice since the administration of kainate to rodents stimulates initially the CA3 region and then generates seizures [37]. Intraperitoneal administration of kainate at 8 mg/kg induced tonic-clonic seizures with loss of the postural tone in mutant mice within 1 h, but not in control mice (Fig. 3A, *P*<0.001, Fisher's exact probability test). Mice of both genotypes showed seizures at a higher dosage of kainate (12 mg/kg), but the latency to the onset of seizures was significantly shorter in mutant mice (Fig. 3B, *P* = 0.03, log-rank test). Neither mutant nor control mice showed seizures after saline-administration. These results suggest that kainate-induced seizure susceptibility was enhanced in mutant mice. Susceptibility to the seizure was comparable between control *GluRζ1* ^flox/flox^ mice and *GluRγ1* ^+/cre^; *GluRζ1* ^+/flox^ mice, indicating that the insertion of the *Cre* gene in one allele of *GluRγ1* locus did not influence the susceptibility.

To monitor the neuronal activity *in vivo*, we employed c-Fos immunohistochemistry. There was little c-Fos immunoreactivity in the hippocampus of both control and mutant mice administrated with saline (*n* = 3, Fig. 3C). Administration of kainate at 8 mg/kg induced strong c-Fos-immunoreactivity in the hippocampus of mutant mice (*n* = 3). In contrast, no significant immunoreactivity was detectable in the hippocampus of kainate-administrated control mice (*n* = 3). Kainate at 12 mg/kg induced strong c-Fos immunoreactivity in both control and mutant mice with seizures, while the number of Fos-immunopositive cells in the hippocampus was significantly smaller in mutant mice than in control mice (*n* = 20 sections from 5 mice). The cellular imaging of neural activity with c-Fos immunohistochemistry confirmed the enhanced seizure susceptibility of mutant mice.

### Histological features of the hippocampal CA3 region

**Figure 4:**
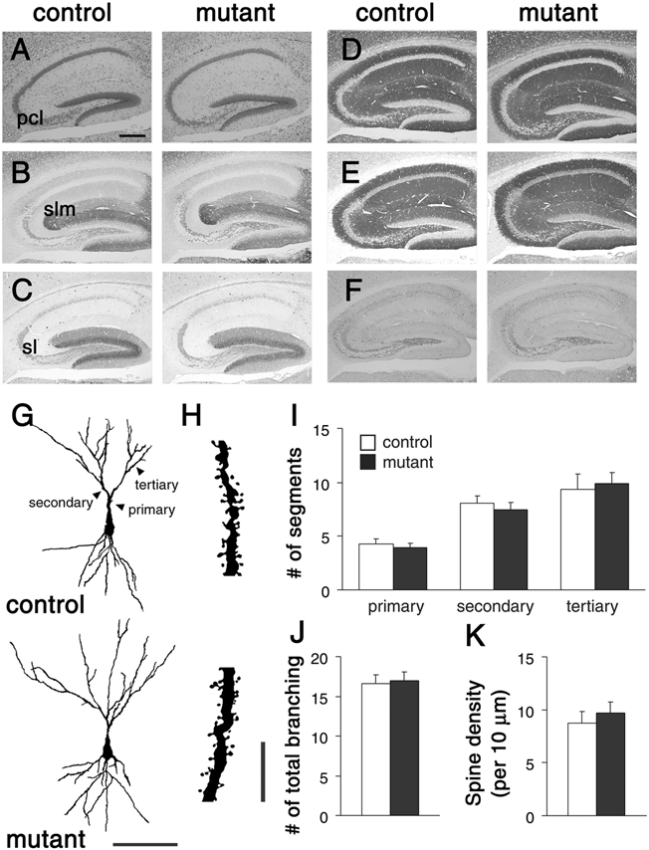
Normal histological organization of the hippocampal region. A, Nissl staining. B, C, Immunoperoxidase staining for VGluT2 (B) and Calbindin (C). D–F, Immunoperoxidase staining for PSD-95 (D), GluRα1 (E), and GAD (F). G, Cytoarchitecture of Golgi-stained CA3 pyramidal neurons. H, Higher magnification of the basal dendritic segment of CA3 pyramidal neuron in (G). I–K, Graphs represents the number of primary, secondary and tertiary dendrites (I), total number of dendritic branching (J), and spine density (K) of CA3 pyramidal neurons. Scale bars: A, 200 µm; G, 100 µm; I, 10 µm. Abbreviations: pcl, pyramidal cell layer; sl, stratum lucidum; slm, stratum lacunosum-moleculare.

Unexpectedly, we found that mutant mice lacking NMDA receptors selectively in CA3 pyramidal neurons became more susceptible to kainate-induced seizures. One obvious possibility is that the ablation of NMDA receptors may disturb the neural wiring of the hippocampal CA3 region, leading to abnormal excitability of the network. We thus examined the histological features of the hippocampal CA3 region in detail. The laminar organization and cellular distribution of the hippocampal CA3 region examined by Nissl staining was indistinguishable between control and mutant mice (Fig. 4A). Immunostaining for vesicular glutamate transporter 2 (VGluT2) and calbindin showed that the afferent terminals from the entorhinal cortex and the dentate gyrus were localized in the stratum lacunosum-moleculare and the stratum lucidum in both control and mutant mice, respectively (Fig. 4B and C).

**Figure 5:**
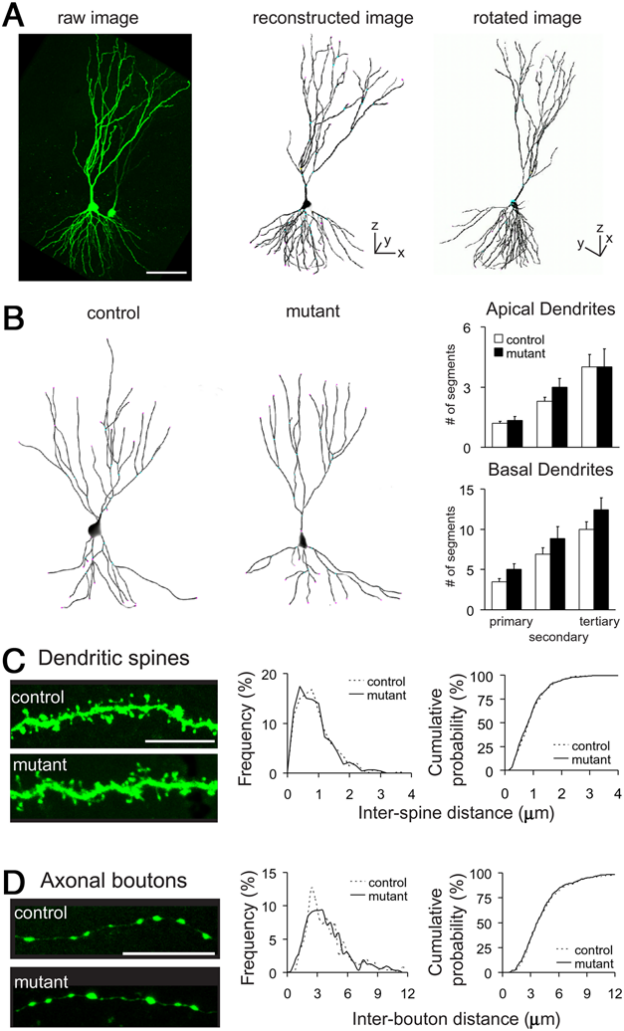
Dendritic branching and distribution of postsynaptic spines and presynaptic boutons in CA3 pyramidal neurons of control and mutant mice. A, Examples of three-dimensional reconstruction using IMARIS and FilamentTracer software. B, Three-dimensional reconstruction of AAV-EGFP-infected CA3 pyramidal neurons. Graphs represent the numbers of primary, secondary and tertiary dendrites of CA3 pyramidal neurons in control (open boxes, *n* = 10–12) and mutant mice (filled boxes, *n* = 6–7). There were no significant differences between control and mutant mice in the numbers of primary (apical, *P* = 0.58; basal, *P* = 0.06; *t*-test), secondary (*P* = 0.13, *P* = 0.21) and tertiary dendrites (*P* = 1.0 *P* = 0.16). C, Tertiary dendritic segments in control (left, top) and mutant (left, bottom) mice. Normalized distribution of inter-spine distances (middle, bin size, 0.1 µm). Cumulative distribution of inter-spine distances (right, same data set). There were no significant differences in inter-spine intervals of CA3 pyramidal neurons between two genotypes (control *n* = 428 from 10 dendrites of 4 mice; mutant, *n* = 459 from 9 dendrites of 4 mice; *P* = 0.74, Kolmogorov-Smirnov test). D, Boutons on the axon in the CA3 stratum radiatum of control (left, top) and mutant (left, bottom) mice. Normalized distribution of inter-bouton distances (middle, bin size, 0.4 µm). Cumulative distribution of inter-bouton distances (right, same data set). There were no significant differences in inter-bouton intervals of CA3 pyramidal neurons between two genotypes (control *n* = 262 from 18 axons of 4 mice; mutant, *n* = 322 from 24 dendrites of 4 mice; *P* = 0.90, Kolmogorov-Smirnov test).

Golgi staining revealed no appreciable differences in dendritic arborization of CA3 pyramidal cells between control and mutant mice (Fig. 4G). There were no significant differences in the numbers of branch points (control, 16.6±1.1, *n* = 8; mutant, 17.0±1.1, *n* = 9; *P* = 0.80; *t*-test) and the primary (control, 4.4±0.5; mutant, 3.8±0.6; *P* = 0.45), secondary (control, 7.8±0.7; mutant, 7.0±0.7; *P* = 0.49) and tertiary dendrites (control, 9.4±1.4; mutant, 9.9±1.0; *P* = 0.76) between two genotypes (Fig. 4I and J). Mean spine density on basal dendrites of CA3 pyramidal cells was also comparable (*n* = 28 dendrites from 3–4 mice, *P* = 0.15) (Fig. 4H and K). Consistent with Golgi staining, fine structures of CA3 neurons visualized by EGFP expression revealed no detectable alteration in terms of dendritic arborization and the distribution of presynaptic axonal boutons and postsynaptic spines (Fig. 5).

Immunoreactivities for postsynaptic proteins, PSD-95 and GluRα1/GluR1, were comparable in the hippocampal CA3 region between the two genotypes (Fig. 4D and E). Distribution of interneurons in the hippocampal CA3 and hilar areas was also indistinguishable as judged by immunostaining for GAD proteins (Fig. 4F), parvalbumin, somatostatin and calretinin. Thus, the histological and cytological organizations of the hippocampal CA3 region were indistinguishable between control and mutant mice.

### Characteristic EEG spikes associated with multiple unit activities in the hippocampal CA3 region of mutant mice

**Figure 6:**
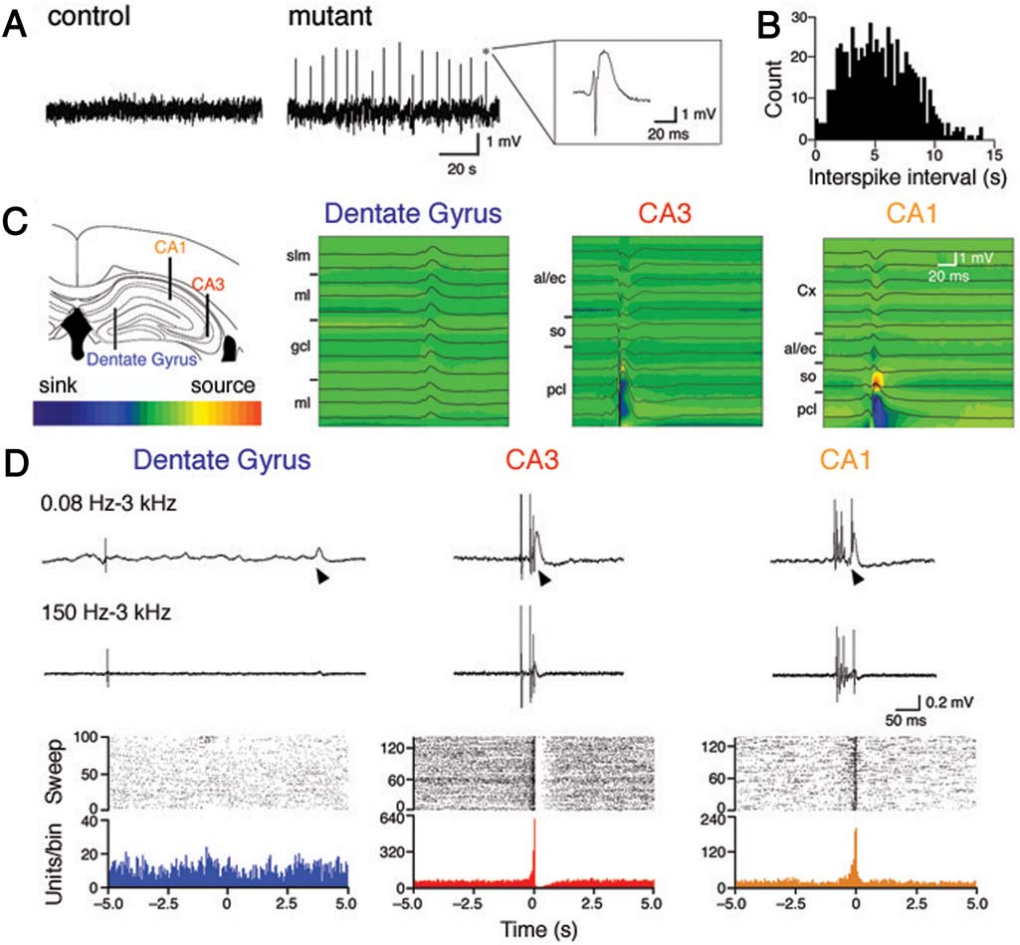
Characteristic large EEG spikes in the hippocampal CA3 region of mutant mice. A, Representative local field potential recordings from the CA3 region. B, Histogram of interspike intervals (bin, 0.25 s). C, Laminar profiles of field potentials and CSD analysis. Recording positions are illustrated on the left. Sinks and sources are indicated by cold and warm colors, respectively. Abbreviations: al/ec, alveus and external capsule; Cx, cortex; gcl, granule cell layer; ml, molecular layer; pcl, pyramidal cell layer; slm, stratum lacunosum-moleculare; so, stratum oriens. D, Wide-band recordings of extracellular activities (top), filtered MUA (middle) and raster plots and peri-event time histograms between MUA (bin, 200 ms) and EEG spikes in the dentate gyrus (left), CA3 (center) and CA1 regions (right). Arrowheads indicate the onset of spikes. MUA were aligned to the onset of spikes (time 0).

Since seizure is produced by synchronous firing of a population of neurons in the brain [38], it is possible that NMDA receptor ablation in the CA3 region may modify hippocampal network oscillations *in vivo*. By recording local field potentials *in vivo* from the hippocampal CA3 region of urethane-anaesthetized mutant mice at the age of postnatal 8 weeks, we found characteristic spikes with large amplitudes (1.5–4.0 mV) (Fig. 6A). These EEG spikes were consistently observed in all 6 mutant mice, but never detected in 7 control mice. The mean firing rate of the spikes (*n* = 136 from 6 mice) was 0.23±0.02 Hz and the distribution of interspike intervals showed a peak at 4.75 s (Fig. 6B).

To investigate the origin of characteristic EEG spikes, we recorded field potentials in various hippocampal regions of mutant mice using a silicon probe with 16 recording sites. Simultaneous recording of a single EEG spike event from the hippocampal CA3 region and surrounding neocortex showed that the amplitude of EEG spikes was largest in the CA3 pyramidal cell layer. EEG spikes reversed their polarity in the CA3 stratum oriens (Fig. 6C). Current source density (CSD) analysis of EEG spikes revealed a current sink in the CA3 pyramidal cell layer, with a source nearby (*n* = 8 from 4 mice). Recording from the cortex and hippocampal CA1 region, spikes reversed their polarity in the CA1 stratum oriens. CSD analyses revealed a large sink in the CA1 pyramidal cell layer (*n* = 8 from 4 mice). On the other hand, EEG spikes recorded from the dentate gyrus showed neither polarity reversal nor sinks in CSD maps (*n* = 8 from 4 mice). These results suggest that characteristic spikes are generated in the pyramidal cell layers of the CA3 and CA1 regions, but not in the dentate gyrus.

Further analysis revealed that the frequency of MUA in the CA3 pyramidal cell layer was enormously high during spike events (Fig. 6D, center). The strong correlation between MUA and EEG spikes was observed in all 4 mutant mice. After EEG spikes, MUA in the CA3 pyramidal cell layer became silent (Fig. 6D, center). MUA in the CA1 pyramidal cell layer were also associated with EEG spikes (Fig. 6D, right) and the association was reproducibly observed in all 4 mutant mice. On the other hand, there was no significant association in the dentate gyrus between MUA and spikes (Fig. 6D, left). The strong association of MUA with EEG spikes in the CA1 and CA3 pyramidal cell layers, but not in the dentate gyrus, together with CA3 pyramidal neuron-selective ablation of NMDA receptors, suggests that characteristic EEG spikes were originated from synchronous firing of CA3 pyramidal neurons and the activity of the CA3 network propagated to the downstream CA1 region.

### Balanced excitatory and inhibitory synaptic transmission

**Figure 7:**
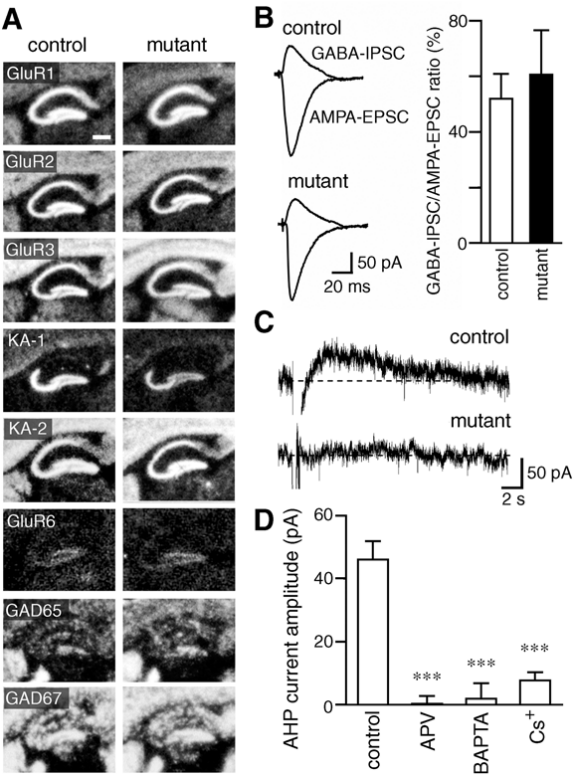
High-frequency stimulation failed to induce slow hyperpolarizing currents in hippocampal CA3 pyramidal neurons of mutant mice. A, X-ray film autoradiography for mRNAs of AMPA receptors, kainate receptors, and GADs. Scale bar, 200 µm. B, Representative traces of AMPA-EPSCs and GABA_A_-IPSCs in the CA3 pyramidal cells. Graph shows the ratio of GABA_A_-IPSCs to AMPA-EPSCs. C, Representative traces of slow hyperpolarizing currents. D, Peak amplitudes of the slow hyperpolarizing currents of the control mice in the absence (control) or presence of D-APV. Those recorded with a BAPTA-containing (BAPTA) or Cs^+^-based internal solution (Cs^+^) are also shown. ***, P<0.001, *t*-test.

Because either enhanced excitation or reduced inhibition can increase the excitability of hippocampal CA3 network, we examined the mRNA levels of excitatory glutamate receptor (GluR) subunits and glutamic acid decarboxylases (GADs) expressed in the hippocampal CA3 region of the mutant mice by *in situ* hybridization (Fig. 7A, Table 1). The *GluRζ1* mRNA was strongly diminished as described above. The reduction of the *GluRγ1* mRNA can be ascribed to the insertion of *cre* into one allele of the *GluRγ1* gene but the *cre* insertion exerted little effect on the kainate-induced seizure susceptibility as described above. There was no significant difference in the *GAD65* mRNA (*P* = 0.08), while the level of *GAD67* mRNA was slightly but significantly reduced in the mutant mice (*P*<0.001). There were no significant differences in hybridization signals of other GluR mRNAs between control and mutant mice.

**Table 1:** Ratios of hybridization signal densities of GluR and GAD mRNAs in the CA3 region to those in the CA1 region.

mRNA	Control	Mutant
*GluRζ1/NR1*	0.86±0.02 (*n* = 10)	0.04±0.01 (*n* = 5)
*GluRα1/GluR1*	0.98±0.02 (*n* = 10)	0.95±0.01 (*n* = 12)
*GluRα2/GluR2*	0.91±0.03 (*n* = 8)	0.89±0.03 (*n* = 11)
*GluRα3/GluR3*	0.88±0.02 (*n* = 10)	0.86±0.02 (*n* = 12)
*GluRγ2/KA-2*	1.16±0.03 (*n* = 9)	1.17±0.02 (*n* = 10)
*GluRβ2/GluR6*	1.11±0.09 (*n* = 8)	1.19±0.04 (*n* = 8)
*GAD65*	1.12±0.05 (*n* = 10)	0.99±0.05 (*n* = 11)
*GAD67*	1.11±0.02 (*n* = 10)	0.94±0.03 (*n* = 12)

Slices were prepared from 3 mice of both genotypes.

Hybridization signal densities of the *GluRγ1/KA-1* mRNA in the CA3 region were 51.5±0.8 (*n* = 10) in control mice and 32.3±0.5 (*n* = 12) in mutant mice.

Basic electrophysiological properties of CA3 pyramidal cells were indistinguishable between two genotypes (resting membrane potential: control, −72.5±0.8 mV, *n* = 32; mutant −73.7±1.0 mV, *n* = 26, *P* = 0.37; input resistance: control, 113.2±5.3 MΩ; mutant, 117.7±7.2 MΩ, *P* = 0.62; membrane capacitance: control, 251.8±9.4 pF; mutant, 250.2±8.0 pF, *P* = 0.90). We then compared GABA_A_-IPSCs in the hippocampal CA3 region, which have been shown to suppress the excitability of the pyramidal cell through postsynaptic inhibition [39]. AMPA-EPSCs were evoked at −80 mV by stimulating afferent fibers in the CA3 stratum radiatum, which should activate both associational/commissural fibers and inhibitory interneurons (and their dendrites and axons), and then GABA_A_-IPSCs were measured with the same stimulus strength at 0 mV in the presence of both the non-NMDA receptor antagonist CNQX and the NMDA receptor antagonist D-APV. The ratio of GABA_A_-IPSCs to AMPA-EPSCs was indistinguishable between the two genotypes (control, 0.52±0.09; mutant, 0.61±0.16; *n* = 12 each; *t*-test, *P* = 0.65) (Fig. 7B). Thus, there was no significant electrophysiological imbalance between AMPA receptor-mediated excitatory and GABA_A_ receptor-mediated inhibitory synaptic transmission in the hippocampal CA3 region.

### High-frequency stimulation failed to induce slow hyperpolarizing currents in hippocampal CA3 pyramidal neurons of mutant mice

In hippocampal CA1 pyramidal neurons, synaptic excitation is followed by an early GABA-mediated hyperpolarization and late AHP mediated by Ca^2+^-dependent K^+^ channels [40]. We thus examined the effect of NMDA receptor ablation on Ca^2+^-dependent K^+^ channels in hippocampal CA3 neurons. At a holding potential of −20 mV, high-frequency stimulation, which should activate both AMPA receptors and NMDA receptors in normal mice, induced slowly decaying outward currents in the pyramidal cells of control mice (Fig. 7C; peak amplitude, 46.1±5.4 pA, *n* = 12). In contrast, such slow outward currents were hardly evoked by the same high-frequency stimulation in mutant mice (Fig. 7C; 0.5±2.1 pA, *n* = 12, *P*<0.001). The outward currents in control mice were abolished by D-APV (Fig. 7D; control, 46.13±5.36 pA, *n* = 13; D-APV, 0.38±2.41 pA, *n* = 12, *P*<0.001), suggesting that NMDA receptors are required for the response. NMDA receptor activation results in influx of Ca^2+^ into postsynaptic cells, which would activate Ca^2+^-dependent K^+^ channels. In fact, inclusion of the Ca^2+^ chelator BAPTA in the internal solution of patch pipettes diminished the outward currents (Fig. 7D; BAPTA, 1.99±4.76 pA, *n* = 7, *P*<0.001). The outward currents were also diminished when recorded with a Cs^+^-based internal solution (Fig. 7D; Cs^+^, 7.74±2.35 pA, *n* = 4, *P*<0.001), suggesting that the currents were mediated by postsynaptic K^+^ channels. Taken together, the slow kinetics and sensitivities to D-APV, BAPTA and Cs^+^ of the outward hyperpolarizing currents suggest that the high-frequency stimulation evokes slow AHP currents [41], [42] mediated by Ca^2+^-activated K^+^ channels, which are activated by Ca^2+^ influx through NMDA receptor channels. These results suggest that the NMDA receptor-slow AHP coupling is diminished in the hippocampal CA3 pyramidal neurons of mutant mice, which may result in enhanced excitability of the CA3 recurrent network as a whole. The coupling between NMDA receptors and AHP currents is found in various regions [34], [43]–[45]. However, the durations of AHP currents observed in our experiment were much longer than those observed in previous studies.

These results with hippocampal CA3-specific NMDA receptor mutant mice raise an intriguing possibility that MDA receptors suppress the excitability of the CA3 recurrent network as a whole by restricting synchronous firing of CA3 neurons, although the possibility cannot be excluded that the enhanced excitability of the mutant mice might be due to subtle cytoarchitectural abnormalities of CA3 pyramidal neurons. To test the possibility, we then examined the effect of NMDA receptor ablation in the CA3 region of the adult brain on hippocampal network oscillations by employing a virus-mediated gene knockout technique [22], [23].

### Ablation of CA3 NMDA receptors in the mature brain also generated characteristic EEG spikes with large amplitudes

**Figure 8:**
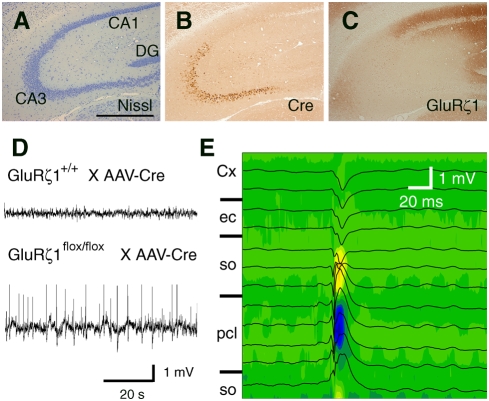
Hippocampal CA3 NMDA receptor ablation in the adult brain also generated characteristic EEG spikes with large amplitudes. A–C, AAV-Cre-mediated ablation of NMDA receptors in the hippocampal CA3 region. Nissl staining (A) and imunohistochemistry for Cre (B) and GluRζ1 (C). Scale bar, 0.5 mm. D, Representative local field potential recordings from the CA3 region. E, Laminar profiles of field potentials and CSD analysis. Recording positions are illustrated on the left. Sinks and sources are indicated by cold and warm colors, respectively. Cx, cortex; ec, external capsule; pcl, pyramidal cell layer; so, stratum oriens.

An adeno-associated viral expression vector for Cre recombinase (AAV-Cre, titer of 5–8×10^10^) was streotaxically microinjected to the hippocampal CA3 region of *GluRζ1* ^flox/flox^ mice at 8–9 weeks old. Immunohistochemical analysis revealed that the infection of AAV-Cre was limited to the hippocampal CA3 region and spread within 40–70% of the region (Fig. 8A–C). Immunoreactivity for GluRζ1 was diminished in the well-demarcated infected CA3 region by 2 weeks after infection (Fig. 8C). Age-matched *GluRζ1* ^+/+^ mice microinjected with AAV-Cre served as controls.

Local field potential recording from the CA3 region showed characteristic EEG spikes with large amplitudes in *GluRζ1* ^flox/flox^ mice 2–3 weeks after AAV-Cre infection (*n* = 5 out of 9 mice) (Fig. 8D). The frequency of large EEG spikes was variable among subjects, which may be related to the variance of AAV-infected regions among animals. No such spike activity was detected in EEG records from the CA3 region of AAV-Cre-infected *GluRζ1* ^+/+^ mice (*n* = 7 out of 7 mice, *P* = 0.02, Fisher's exact probability test) (Fig. 8D). CSD analysis revealed the sink in the pyramidal cell layer of the CA3 region and the sources in neighboring stratum oriens (Fig. 8E, *n* = 8 spikes). Thus, the ablation of CA3 NMDA receptors induced by AAV-Cre infection in the adult brain also resulted in the generation of characteristic EEG spikes.

### Pharmacological blockade of CA3 NMDA receptors enhanced the susceptibility to kainate-induced seizure

**Figure 9:**
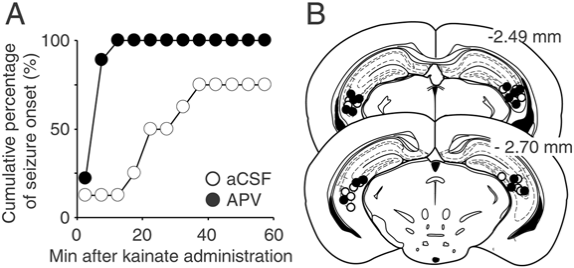
The pharmacological blockade of CA3 NMDA receptors increased the susceptibility to kainate-induced seizures. A, Cumulative curves for the onset of seizure. B, Illustration of the injection sites of APV (filled) and aCSF (open). Numbers represent distance (mm) of the section relative to the bregma landmark.

We finally examined the seizure susceptibility of wild-type mice by focal injection of a competitive NMDA receptor antagonist, APV. We bilaterally injected 30 mM APV or aCSF into the hippocampal CA3 region of C57BL/6N mice at postnatal 8–10 weeks. About 20–30 minutes later, the animals were intraperitoneally administrated with the convulsive dose of kainate (30 mg/kg) [46]. Kainate-induced tonic-clonic seizures with loss of the postural tone appeared within 1 h in both groups of mice (*n* = 8 each; *P* = 0.23, Fisher's exact probability test) (Fig. 9). However, the latency to the onset of seizures was significantly shorter in mice injected with APV (*n* = 8; *P* = 0.0044, Log-rank test). Thus, the focal blockage of CA3 NMDA receptors also enhanced the susceptibility to kainate-induced seizure.

## Discussion

Here, we generated hippocampal CA3 pyramidal neuron-specific NMDA receptor mutant mice on the C57BL/6N genetic background. The expression of the *GluRζ1* mRNA was comparable between mutant and control mice at P1 but strongly decreased in mutant mice at P7. The significant expression of GluRζ1 protein, though reduced, was found in the CA3 region at P7 but diminished to a negligible level by P14. We found that the mutant mice lacking NMDA receptors in the hippocampal CA3 pyramidal neurons showed enhanced susceptibility to kainate-induced seizures. This observation was rather unexpected since NMDA receptor-mediated LTP was implied to contribute to the generation of synchronous network activity by *in vitro* studies [14], [15]. We found that characteristic EEG spikes with large amplitude were generated by the ablation of NMDA receptors in CA3 pyramidal neurons. Strong association of MUA with the characteristic EEG spikes in the CA3 pyramidal cell layer of mutant mice suggests that the CA3 NMDA receptor ablation increases the synchronous network activity possibly by affecting the firing pattern of CA3 neurons. In contrast, CA1 region-specific ablation of NMDA receptors appeared to hardly affect EEG *in vivo* [47]. NMDA receptor antagonists have minimal effects on basal synaptic transmission but completely block the generation of long-term potentiation in the CA1 region *in vitro* [48]–[50]. Hence, NMDA receptors in the CA1 region are not considered to be involved in spontaneous network activity. The difference in the neural wiring pattern such as the abundance of recurrent networks may underlie the different effects of NMDA receptor ablation in the hippocampal CA1 and CA3 regions on network activity. Our results raise an intriguing possibility that NMDA receptors may suppress the excitability of the CA3 network as a whole *in vivo*.

It is possible that the ablation of NMDA receptors may disturb the neural wiring of the hippocampal CA3 region, leading to abnormal excitability of the network. It is well known that the NMDA receptor plays a role in the activity-dependent refinement of synaptic connections and neural pattern formation [51]–[54]. Chronic blockade of NMDA receptors in hippocampal slice cultures during the first two weeks of postnatal development leads to a substantial increase in synapse number and results in a more complex dendritic arborization of CA1 pyramidal cells [31]. The activity blockade in hippocampus during postnatal 2–3 weeks by tetrodotoxin infusion produced both behavioral and electrographic seizures 2 weeks after the infusion [55] and the increase in the density of axonal varicosities and postsynaptic AMPA receptor GluR1 and NMDA receptors [56]. Thus, reduced neuronal activity during development might potentially enhance the excitability. However, the cytoarchitecture was indistinguishable between control and mutant mice at P21–23. There were no detectable differences in the dendritic branching and the density of axonal boutons and dendritic spines between control and mutant mice at P21–23. The sustained expression of NMDA receptor proteins at least by P7 in mutant mice may support the development of CA3 pyramidal neuron cytoarchitectures. An alternative possibility is that the excitability of the CA3 network may be suppressed by NMDA receptor-mediated signaling. No significant differences were detectable in the basic membrane properties and balance between excitatory and inhibitory synaptic transmission between control and mutant mice. At synapses, activation of NMDA receptors evokes excitatory postsynaptic potential on the CA3 pyramidal neurons *in vitro* [57]. However, the enhancement of the kainate-induced seizure susceptibility and the emergence of characteristic EEG spikes associated with MUA in the mutant mice can be hardly explained if major roles of NMDA receptors would be simply mediating and strengthening the excitatory transmission at the commissural/associational synapses. Besides excitatory transmission and its enhancement, NMDA receptors may mediate diverse suppressive signals including spike-timing dependent long-term depression [58], LTP of slow GABA-IPSCs [59], the increase in I_h_ currents [60], and coupling with K^+^ channels [34], [43]–[45]. Thus, it is possible that NMDA receptor signaling may suppress the excitability of the CA3 network *in vivo*, although the possibility cannot be excluded that the enhanced excitability of the mutant mice might be due to subtle developmental abnormalities of CA3 pyramidal neurons.

We thus examined whether the excitability of the CA3 network is enhanced by ablation of NMDA receptors in the adult brain with a virus-mediated gene knockout technique [22], [23]. We found that EEG spikes with large amplitude were generated by focal ablation of NMDA receptors in the CA3 region of adult mice by AAV-Cre infection. The frequency of large EEG spikes was variable among subjects, which may be related to the variance of AAV-infected regions among animals. Furthermore, the blockade of NMDA receptors by focal injection of APV into the hippocampal CA3 region enhanced the susceptibility to kainate-induced seizures. These results suggest that NMDA receptors control negatively the excitability of the hippocampal CA3 recurrent network as a whole *in vivo* by restricting synchronous firing of CA3 neurons, although the mechanism remains to be solved. Since slow AHP currents are involved in accommodation of action potential discharge of CA1 pyramidal neurons [40], it is possible that the frequency of action potentials may increase in a mutant CA3 pyramidal neuron where NMDA receptor-AHP coupling is eliminated. Prolonged discharges of CA3 pyramidal neurons might increase the chance of their synchronous firing, leading to the enhancement of the excitability of the CA3 network as a whole. Interestingly, Colgin et al. reported that blockade of NMDA receptors enhanced spontaneous sharp waves in rat hippocampal slices [61], supporting the idea that activation of NMDA receptors can serve to dampen the excitation of sharp waves. On the other hand, studies through computational models showed that when recurrent networks with conductance delays exhibit population bursts, spike-timing-dependent plasticity (STDP) rules exert a strong decoupling force that desynchronizes activity [58]. Thus, elimination of NMDA receptor-dependent STDP might enhance synchronization in CA3 recurrent networks. One or combination of such NMDA receptor-mediated suppressive signals [34], [43]–[45], [58]–[60] might underlie the regulation of CA3 network excitability. The NMDA receptors in the hippocampal CA3 region are implied in rapid acquisition and recall of associative memory as well as paired associate learning [11]–[13]. These functions may be mediated not only by the plasticity at synapses but also by the NMDA receptor-mediated neural network oscillation.
